# Mind the gap – Small bowel obstruction due to preperitoneal herniation following laparoscopic inguinal hernia repair – A case report

**DOI:** 10.1016/j.ijscr.2021.106532

**Published:** 2021-10-18

**Authors:** Andreas Thalheimer, Rene Vonlanthen, Silviya Ivanova, Christoforos Stoupis, Marco Bueter

**Affiliations:** a Department of Visceral and Transplantation Surgery, University Hospital of Zürich, Zürich, Switzerland; b Department of Surgery, Spital Männedorf, Männedorf, Switzerland; c Department of Radiology, Spital Männedorf, Männedorf, Switzerland

**Keywords:** Case report, Inguinal hernia repair, Preperitoneal herniation, Small bowel obstruction

## Abstract

**Introduction and importance:**

Inguinal hernia repair is a very frequent operation in general and visceral surgery worldwide. The laparo-endoscopic approaches such as TAPP have gained increasing acceptance among specialists and many consider them as standard of care due to perioperative safety and excellent postoperative results. Knowledge of specific complications after minimally invasive inguinal hernia surgery, however, is important for the successful management of these patients.

**Case presentation:**

We herein present the case of a 75-year-old female patient who electively underwent laparoscopic repair of combined inguinal and femoral hernia. During the postoperative course a small bowel obstruction occurred requiring emergency re-laparoscopy revealing a preperitoneal herniation of small bowel through a peritoneal defect.

**Clinical discussion:**

Small bowel obstruction due to preperitoneal herniation of small bowel through a peritoneal defect after laparoscopic hernia repair is extremely rare. In such cases, emergency laparoscopic revision is necessary to avoid bowel ischaemia. Adequate closure of the peritoneum during the primary procedure along with the necessary attention to detail seems mandatory to avoid preperitoneal herniation after TAPP.

**Conclusion:**

Inadequate peritoneal closure after TAPP may lead to preperitoneal herniation of the small bowel leading to postoperative intestinal obstruction. All hernia surgeons should be aware of this rare, but potentially life-threatening complication and should close all peritoneal defects with greatest care and accuracy.

## Highlights

• Laparoendoscopic techniques are standard treatment for inguinal hernia repair
• Specific complications due to the intraabdominal approach can occur in laparoscopic TAPP
• Herniation of small bowel through peritoneal gaps following laparoscopic TAPP can cause serious postoperative complication
• The early detection of this complication is important to avoid a threatening postoperative course
• Careful closure of all peritoneal gaps is critical to avoid preperitoneal herniation

## 1 Introduction

Laparo-endoscopic techniques like the transabdominal preperitoneal patch plasty (TAPP) have fundamentally changed the surgical treatment of inguinal hernias and are characterized by less post-operative pain, faster recovery, a shorter hospital stay, better cosmesis, easier repair of recurrent hernias, and the ability to treat bilateral hernias concurrently when compared to conventional techniques such as the Lichtenstein- or Shouldice procedure [1]. The postoperative complication rate after laparoendoscopic techniques is rather low and includes seroma formation, visceral injury, chronic pain and testicular complications. As increasing numbers of TAPP procedures are being performed worldwide, uncommon complications become more frequent and need to be considered in the peri- and postoperative management. Small bowel obstruction (SBO) represents such a rare complication after TAPP and has been reported to occur in approximately 0.1–0.3% of cases [2], [3]. Among the potential causes of SBO after TAPP is a preperitoneal herniation of small bowel through a peritoneal defect [4]. Other causes are herniation at the trocar site as well as obstruction due to postoperative adhesions [5], [6].

Herein, we report the case of a 75-year-old female with a preperitoneal herniation of small bowel leading to a mechanical obstruction shortly after TAPP for treatment of a combined inguinal and femoral hernia. This case report has been written in line with the SCARE Criteria [7].

## 2 Presentation of case

A 75-year-old female presented with a swelling in the right groin that had been present for several months. The patient felt disturbed by the increasing swelling. Sonography and low dose abdominal CT confirmed the diagnosis of a right sided inguinal hernia. The indication for inguinal hernia repair by a laparoscopic TAPP procedure with mesh reinforcement was made. The procedure was performed by an experienced hernia surgeon. Intraoperatively, there was a combined inguinal and femoral hernia on the right side. TAPP was uneventful and performed according to standardized steps with a 12x17cm mesh augmentation (BARD 3DMax® Lightmesh). The peritoneal flap was closed using a barbed wire (Ethicon, Stratafix™ 3-0).

**Fig. 1:**
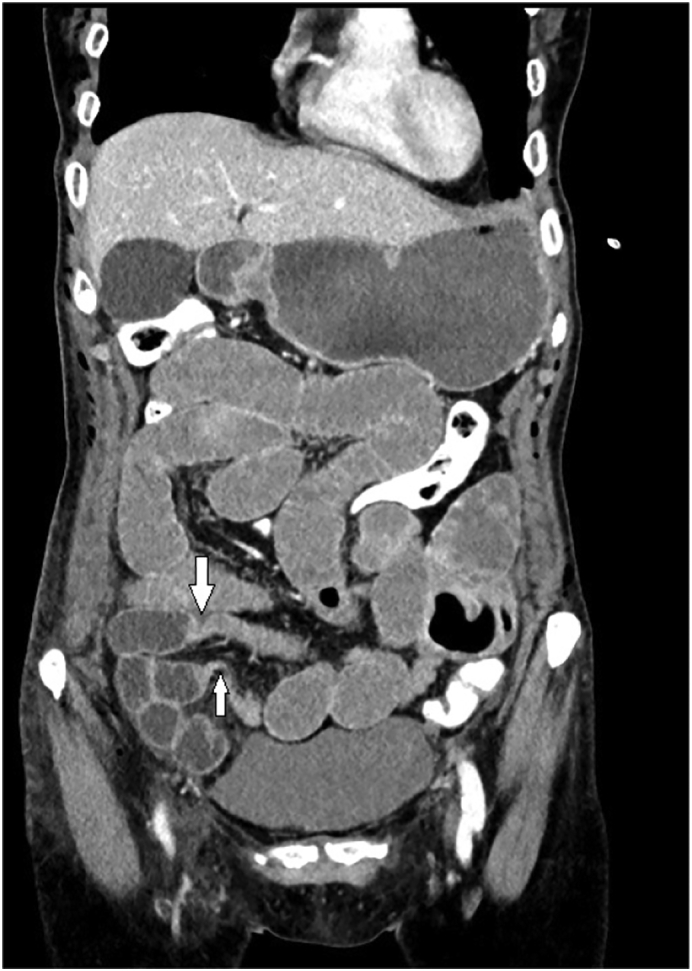
CT Abdomen with evidence of a small bowel obstruction at two contiguous points (white arrows) by means of closed loop obstruction. See also dilated small bowel loops proximal to closed loop site.

**Fig. 2:**
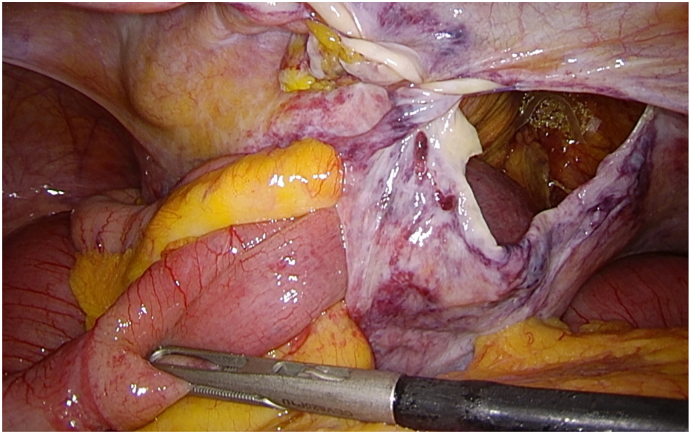
Intraoperative view with preperitoneal herniation of a small bowel loop through a peritoneal gap with secondary dehiscence of the suture of the peritoneal flap.

On postoperative day 2, the patient presented with abdominal distention, sensation of bloating and nausea. A conventional x-ray of the abdomen in upright position revealed moderately dilated loops of the small intestine (< 3 cm) and no differential air-fluid levels. Based on the assumption of postoperative paralysis of the small bowel, a conservative therapy was initiated. On postoperative day 3, the condition of the patient worsened with increasing nausea and abdominal pain. Abdominal CT was performed and demonstrated a closed loop small bowel obstruction (Fig. 1) recommending emergency re-laparoscopy. During re-laparoscopy, the origin of the mechanical obstruction was found to be a loop of the small bowel herniated into the preperitoneal space through a peritoneal defect (Fig. 2). The suture line of the peritoneal flap was also dehiscent most likely due as a consequence of the herniated bowel. The loop of the small intestine could be repositioned without problems and showed no signs of ischemic or hemorrhagic infarction. The preperitoneal mesh was still correctly positioned and no repositioning was necessary. After the peritoneum was closed again with a barbed wire (Ethicon, Stratafix™ 3-0), the operation could be terminated laparoscopically. The further postoperative course was uneventful, so that the patient was discharged on postoperative day 5.

## 3 Clinical discussion

In this case report, we describe the rare complication of small bowel obstruction due to preperitoneal herniation of a loop of small intestine following elective laparoscopic TAPP for inguinal hernia repair.

Laparoscopic techniques for treating inguinal hernia have revolutionized hernia surgery worldwide. The surgical advantages of this technique are obvious: clear representation of the anatomy, treatment of combined inguinal and femoral hernia as well as the possibility of uncomplicated bilateral inguinal hernia treatment [8]. The reduction in postoperative morbidity and the decrease in the rate of chronic inguinal pain syndromes in the long run further contribute to the widespread acceptance of laparoendoscopic techniques [9].

However, the fact that an abdominal operation is used to treat an extra-abdominal problem leads to some special features with regard to the peri- and postoperative complication spectrum. As with all laparoscopic surgery, injury to parts of the bowel or large intra-abdominal blood vessels is, in principle, possible, although very rare [3]. These complications can be avoided by strictly following the recommendations for safe placement of the pneumoperitoneum and strict indication in the case of previous abdominal operations [10].

However, the necessity of opening the preperitoneal space by incising the parietal peritoneum and closing the peritoneum after hernia repair can lead to specific problems.

The group around Dietz et al., for example, demonstrated a case of small bowel volvulus with subsequent small bowel obstruction as a result of the adherence of the small bowel to the insufficiently shortened thread remnant of a barbed wire, which was used to close the peritoneal flap [11]. As the use of this suture material has become standard nowadays, attention must be paid to sufficient shortening at the peritoneal flap.

Some case reports with preperitoneal herniation of the small bowel and subsequent small bowel obstruction have been published [4], [12]. The cause of herniation into the preperitoneal space may be caused by an insufficient closure of the peritoneal flap with loosening of the barbed wire and successive displacement of the small bowel. As in this case, however, the small bowel may also herniate through an inadequately closed peritoneal defect that may occur during the preparation of the peritoneum due to a very delicate tissue texture or also due to difficult preparation caused by postoperative adhesions of previous operations. The resulting small bowel obstruction usually leads to rapid onset of postoperative ileus symptoms.

A randomized comparison of suture and stapler technique for peritoneal closure with respect to patient outcomes in TAPP, favors the suture technique in terms of postoperative pain reduction [13]. However, no statements can be made about complications like preperitoneal herniation due to the rarity of the event.

Considering the published case reports, the following technical advice seems important for avoiding small bowel obstruction after laparoscopic hernia repair:

- Closure of all peritoneal defects. If a barbed wire is used, the exact shortening of the remaining thread is necessary and the independent loosening of the thread must be avoided.
- Any gaps in the peritoneum besides the suture line of the peritoneal flap that occur during the preparation must be closed without exception.
- If small bowel obstruction occurs in the short-term postoperative course after laparoscopic TAPP, preperitoneal herniation of the small bowel should be considered.

## 4 Conclusion

Small bowel obstruction after laparoscopic inguinal hernia repair may be caused by preperitoneal displacement of the small bowel through a peritoneal defect with consecutive mechanical small bowel obstruction. Accurate closure not only of the peritoneal flap, but also of all other defects in the peritoneum, is absolutely mandatory to avoid this complication and emergency re-laparoscopies.

## Sources of funding

None.

## Ethical approval

N/A.

## Consent

Written informed consent was obtained from the patient for publication of this case report and accompanying images. A copy of the written consent is available for review by the Editor-in-Chief of this journal on request.

## Research registration

N/A.

## Guarantor

Andreas Thalheimer.

## Provenance and peer review

Not commissioned, externally peer-reviewed.

## CRediT authorship contribution statement

Andreas Thalheimer: Conceptualization, Writing Original draft, Writing& Editing Review.

Rene Vonlanthen: Conceptualization, Review of Original draft.

Silviya Ivanova: Conceptualization, Review Original Draft.

Christoforos Stoupis: Conceptualization, Review Original Draft.

Marco Bueter: Conceptualization, Writing Original Draft, Writing & Editing Review.

## Declaration of competing interest

None.

## References

[1] Kockerling F., Simons M.P. (2018). Current concepts of inguinal hernia repair. Visc. Med..

[2] Kapiris S.A., Brough W.A., Royston C.M., O'Boyle C., Sedman P.C. (2001). Laparoscopic transabdominal preperitoneal (TAPP) hernia repair. A 7-year two-center experience in 3017patients. Surg. Endosc..

[3] Sartori A., De Luca M., Noaro G., Piatto G., Pignata G., Di Leo A., Lauro E., Andreuccetti J. (2021). Rare intraoperative and postoperative complications after transabdominal laparoscopic hernia repair: results from the Multicenter Wall Hernia Group Registry. J. Laparoendosc. Adv. Surg. Tech. A.

[4] Zou Z., Zhu Y., Wang F., Cao J., Liu Y., Yang H., Wang M. (2021). Preperitoneal herniation as a complication of tansabdominal preperitoneal patch plasty: a report of two cases. BMC Surg..

[5] Zheng L., Yin X., Liu H., Wang S., Hu J. (2021). Case report: small bowel obstruction owing to self-anchoring barbed suture device after TAPP repair. Front. Surg..

[6] Harriott C.B., Dreifuss N.H., Schlottmann F., Sadava E.E. (2021). Incidence and risk factors for umbilical trocar site hernia after laparoscopic TAPP repair. A single high-volume center experience. Surg. Endosc..

[7] Agha R.A., Franchi T., Sohrabi C., Mathew G., Kerwan A., Group S (2020). The SCARE 2020 guideline: updating consensus Surgical CAse REport (SCARE) guidelines. Int. J. Surg..

[8] HerniaSurge G. (2018). International guidelines for groin hernia management. Hernia.

[9] Berger D. (2016). Evidence-based hernia treatment in adults. Dtsch. Arztebl. Int..

[10] Bittner R. (2017). Evidence-based TAPP technique. Chirurg.

[11] Filser J., Reibetanz J., Krajinovic K., Germer C.T., Dietz U.A., Seyfried F. (2015). Small bowel volvulus after transabdominal preperitoneal hernia repair due to improper use of V-loc barbed absorbable wire - do we always "read the instructions first"?. Int. J. Surg. Case Rep..

[12] Narayanan S., Davidov T. (2018). Peritoneal pocket hernia: a distinct cause of early postoperative small bowel obstruction and strangulation: a report of two cases following robotic herniorrhaphy. J. Minim. Access Surg..

[13] Oguz H., Karagulle E., Turk E., Moray G. (2015). Comparison of peritoneal closure techniques in laparoscopic transabdominal preperitoneal inguinal hernia repair: a prospective randomized study. Hernia.

